# Spectrum of Hereditary Ataxia in Omani Children

**DOI:** 10.3390/jcm15187216

**Published:** 2026-09-17

**Authors:** Amal Al-Habsi, Khalid Al-Thihli, Eiman Al-Ajmi, Amna Al-Futaisi, Abdullah Al-Badi, Fatema Al-Amrani

**Affiliations:** 1 Pediatric Residency Program, Oman Medical Specialty Board, Muscat 130, Oman; r21142@resident.omsb.org (A.A.-H.); r25153@resident.omsb.org (A.A.-B.); 2 Department of Genetics, Sultan Qaboos University Hospital, University Medical City, Muscat 123, Oman; 3 College of Medicine and Health Science, Sultan Qaboos University, Muscat 123, Oman; ealajmi@squ.edu.om; 4 Department of Radiology and Molecular Imaging, Sultan Qaboos University Hospital, University Medical City, Muscat 123, Oman; 5 Department of Child Health, College of Medicine and Health Sciences, Sultan Qaboos University, Muscat 123, Oman; amnaf@squ.edu.om; 6 Pediatric Neurology Unit, Department of Child Health, Sultan Qaboos University Hospital, University Medical City, Muscat 123, Oman

**Keywords:** hereditary ataxia, autosomal recessive ataxia, autosomal dominant ataxia, ataxia telangiectasia, spinocerebellar ataxia, magnetic resonance imaging

## Abstract

**Background/Objectives:** Hereditary ataxias are a genetically and phenotypically heterogeneous group of neurodegenerative disorders that often pose diagnostic challenges. This study characterized the spectrum of hereditary ataxias encountered at our institution and identified phenotypic patterns that may facilitate early recognition and diagnosis. **Methods:** A retrospective cohort study included patients younger than 18 years presenting with ataxia at Sultan Qaboos University Hospital between January 2012 and January 2022. Medical records were reviewed for demographic, clinical, molecular genetics, and neuroimaging data. Ataxia was classified as acquired or hereditary. Hereditary cases were identified based on clinical features and family history and further divided into genetically confirmed and non-genetically confirmed. Selected clinical variables were evaluated for their impact on the diagnostic yield of hereditary ataxia. Data analysis was performed using SPSS version 29, with a significance level set at *p* < 0.05. **Results:** A total of 132 patients were identified with ataxia, and 70 met the diagnostic criteria for hereditary ataxia. Among the 70 patients, 46 (65.7%) had autosomal recessive ataxia, 9 (12.9%) had apparently sporadic ataxia for whom autosomal recessive inheritance could not be excluded, and 4 (5.7%) had autosomal dominant ataxia. Ataxia telangiectasia was the most frequent inherited ataxia in our cohort, 12/70 (17%), followed by Charlevoix-Saguenay spastic ataxia, 5/70 (7.1%). There were no significant differences in clinical characteristics or neuroimaging findings between the two groups, including peripheral neuropathy. **Conclusions:** Ataxia telangiectasia represents the most common form of hereditary ataxia in our cohort. Peripheral neuropathy was more frequently observed in genetically confirmed patients, but this difference was not statistically significant. Early recognition and molecular diagnosis of potentially treatable neurometabolic and movement disorders remain important for timely management and prognosis.

## 1. Introduction

Hereditary cerebellar ataxias (HCAs) in childhood are a clinically, genetically, and pathologically heterogeneous group of disorders with almost 200 known genes, 90 of which primarily cause recessive ataxia [1,2]. HCAs are characterized by progressive incoordination in association with action tremor, dysarthria, and nystagmus [3]. Furthermore, some types of HCA may have extra-cerebellar manifestations that include peripheral neuropathy, cognitive and behavioral issues, epilepsy, pyramidal and extrapyramidal signs, or eye abnormalities, i.e., retinopathy, optic atrophy, and supranuclear palsies [4].

These groups of disorders are disabling and carry a high rate of morbidity and mortality [5]. HCAs can be classified using different classification systems—the most widely used is the mode of inheritance. In this classification, ataxia can be classified as autosomal dominant cerebellar ataxias (ADCA), autosomal recessive cerebellar ataxias (ARCA), X-linked ataxias, and maternally inherited ataxias as part of the mitochondrial disease phenotype [6]. Childhood-onset ataxia is usually of the autosomal recessive type, and its most common subtypes are Friedreich ataxia (ATX-*FXN*), ataxia-telangiectasia (ATX-*ATM*), and ataxia with oculomotor apraxia type 1 and type 2 [7]. Family history, physical examination, neuroimaging, laboratory investigations such as alpha-fetoprotein (AFP), albumin, and cholesterol levels, and molecular genetic testing are important components of the diagnostic evaluation of HCA [1].

The scarcity of studies exploring the prevalence and genetic spectrum of hereditary cerebellar ataxias (HCAs) in the Middle East and North Africa (MENA) region indicates a need for large-scale research to enhance an understanding of the underlying genetics and to develop region-specific diagnostic approaches suited to this unique population. The Omani population, characterized by a high rate of consanguinity, provides a unique context in which recessively inherited disorders, including ataxia, are likely to be more prevalent [8,9]. However, data on the incidence, prevalence, and genotype–phenotype correlations of HCA in Oman remain scarce. Therefore, this retrospective study was conducted to examine the clinical and genetic landscape of this heterogeneous group of disorders and to address the existing gaps of knowledge related to this population.

This study was conducted to (a) describe the spectra of hereditary ataxia seen at Sultan Qaboos University Hospital (SQUH), (b) delineate phenotypic features that could aid in early diagnosis, (c) compare our findings to what has been published in the literature for other ethnic groups when available, and (d) calculate the diagnostic yield (DY) of genetic testing, including whole exome sequencing (WES) for a diagnosis of hereditary cerebellar ataxia.

## 2. Materials and Methods

### 2.1. Patients and Study Design

This study is a retrospective chart review of patients under the age of 18 who presented with ataxia at the tertiary referral facility SQUH in Muscat, Oman, between January 2012 and January 2022. We identified patients using the Hospital Information System (HIS) by searching for the keywords ataxia, cerebellar ataxia, and hereditary ataxia. All patients underwent a thorough review to confirm they met the definition of ataxia and to identify those who met the inclusion criteria [3].

The inclusion criteria for this study were as follows: participants with an age at symptom onset of less than 18 years and those who met the definition of hereditary ataxia. To define hereditary ataxia, we adapted the diagnostic criteria proposed by Jayadev et al. (2013) requiring the presence of cerebellar ataxia along with at least one of the following: (a) an ataxia phenotype consistent with a recognizable heritable condition or etiology, (b) two or more affected individuals within a family, allowing the pedigree to suggest a plausible mode of inheritance, (c) an ataxia phenotype accompanied by additional features or organ involvement not explained by an acquired condition, (d) absence of an established acquired etiology, or (e) consanguineous parents with an affected child whose ataxia could not be attributed to an acquired cause, even in the absence of another affected relative [3]. Exclusion criteria included patients with a confirmed acquired cause of ataxia, individuals with onset of symptoms after 18 years of age, and those who did not fulfill the above diagnostic criteria. The term “hereditary ataxia” was used as a clinical inclusion criterion and was not restricted to disorders classified as primary hereditary ataxias; therefore, the cohort included genetic disorders in which ataxia may occur as part of a broader neurodevelopmental, metabolic, neurodegenerative, or multisystem phenotype.

### 2.2. Categorization of Patients

We included all patients who met the inclusion criteria for the study. For each patient, we collected clinical data, including age at symptom onset, age at data collection, sex, and the presence of other clinical features, such as developmental delay, neuroregression, seizures, peripheral neuropathy, and eye findings on examination when available. Also, for each patient, we reviewed and collected neuroimaging and molecular genetics findings. A pathogenic variant causing hereditary cerebellar ataxia (HCA) in two or more Omani families that were not related by blood or geographical location was presumed to be the founder variant when it was rarely encountered in the local genetic database, but this presumption was made only for the purpose of guiding genetic evaluation of further families, with the understanding that further confirmation was not performed in this study. For the recurrent hereditary ataxias in our cohort, we further assessed clinical features that might aid in an earlier diagnosis. We classified our studied population into two groups: those who had genetically confirmed HCA and a second group with non-genetically confirmed HCA. We further classified patients with genetically confirmed HCA into seven groups based on the inheritance pattern predicted by family pedigrees: ADCA, ARCA, X-linked, mitochondrial, apparently sporadic (AS), X-linked versus AS, and AS versus AR. Patients with non-genetically confirmed ataxia were evaluated separately and were further categorized according to the pattern of inheritance. Further description of both groups was provided based on the clinical presentation, presence of extra-cerebellar manifestations, neuroimaging, and the molecular genetics findings.

### 2.3. Neuroimaging

A neuroradiologist (E.A.) reviewed the neuroimaging of all the patients. The magnetic resonance imaging (MRI) findings were categorized as adapted from Perucca et al. [10]. On each scan, the cerebellar morphology was classified as normal, showing cerebellar atrophy or hypoplasia, and cerebellar signals were evaluated as normal, demonstrating T2/FLAIR hyperintensity involving the cortex, white matter, or deep nuclei. Brainstem morphology was categorized as normal, malformed, or atrophic, with the signal being characterized as normal or showing T2/FLAIR hyperintensity. Cerebral hemispheres were assessed for the presence of atrophy. The supratentorial white matter signal was categorized as normal or demonstrating T2/FLAIR hyperintensities involving the deep and periventricular white matter, subcortical white matter, or corticospinal tracts or with diffuse white matter involvement. Basal ganglia were evaluated for the presence of atrophy and/or signal abnormalities. In addition, scans were reviewed for the presence of calcifications, and spinal cord morphology was assessed for atrophy. The definitions of cerebellar atrophy and cerebellar hypoplasia or dysplasia were adapted from Vedolin et al. [11].

### 2.4. Genetic Testing

Patients were eligible for inclusion based on the predefined study inclusion criteria for hereditary cerebellar ataxia (HCA). The results of molecular genetic testing performed for patients with suspected HCA were reviewed. Genetic testing varied according to clinical presentation and family history and included whole-exome sequencing (WES), an HCA multigene panel, targeted variant analysis, or microarray. Variant pathogenicity was assessed according to the American College of Medical Genetics and Genomics (ACMG) variant classification guidelines [12]. Variants initially classified as variants of uncertain significance (VUS) were subsequently re-evaluated by the local molecular genetics and genomics laboratory, taking into consideration the patient’s phenotype, frequency in the local general population, confirmed de novo inheritance in cases with an autosomal dominant (AD) diagnosis, family segregation analysis, and additional evidence supporting pathogenicity, including functional studies where available.

Molecular genetic testing was performed at several external accredited clinical laboratories, including Fulgent Genetics, Medical Neurogenetics, Breda Genetics, and Centogene, according to their respective validated testing methodologies. The specific next-generation sequencing (NGS) platforms varied between laboratories and over the study period; however, platform-level information was not systematically recorded in our retrospective dataset and therefore could not be consistently reported. Family pedigrees were reviewed to determine the presumed mode of inheritance. When there was no family history of similar disease or consanguinity and the proband was the only affected family member, the condition was classified as apparently sporadic (AS). However, autosomal recessive (AR) inheritance could not be excluded in small or non-extended families, and X-linked inheritance could not be excluded in male patients.

### 2.5. Ethical Approval

Ethical approval was obtained from the Medical Research Ethics Committee at SQUH (REF.NO. SQU-E/068/2022).

### 2.6. Data Analysis

This is a study describing the clinical features, neuroimaging findings, and molecular genetic testing of our cohort of patients. Evaluation and analysis of the obtained data were performed using the Statistical Package for the Social Sciences (SPSS) software, version 29, IBM, Chicago, IL, USA. The data are presented as the number and percentage of patients. Continuous variables were summarized using means, standard deviations, medians, and ranges, as appropriate. Categorical variables were summarized as a number and percentage and compared between the genetically confirmed and non-genetically confirmed groups using Fisher’s exact test due to the small sample size and low expected cell frequencies. All tests were two-sided, and a *p*-value < 0.05 was considered statistically significant.

### 2.7. Artificial Intelligence Tools

Generative artificial intelligence (GenAI) tools were used during manuscript preparation to assist with language refinement and organization. ChatGPT (OpenAI, version GPT-4) was used to assist with improving the clarity, readability, grammar, and organization of the manuscript. QuillBot (version 6.0) was used to assist with language refinement and paraphrasing to improve readability. Perplexity (version 1.8, powered by Claude 2) was used to assist with literature searching and to identify potentially relevant scientific publications and sources. All literature identified through these tools was independently reviewed by the authors, and relevant information was verified against the original publications before inclusion in the manuscript. GenAI tools were not used to generate or modify the study data, perform statistical analyses, generate research results, or make final scientific interpretations. All GenAI-assisted content was critically reviewed and revised by the authors, who take full responsibility for the accuracy, originality, and integrity of the final manuscript.

## 3. Results

### 3.1. Patients

**Figure 1:**
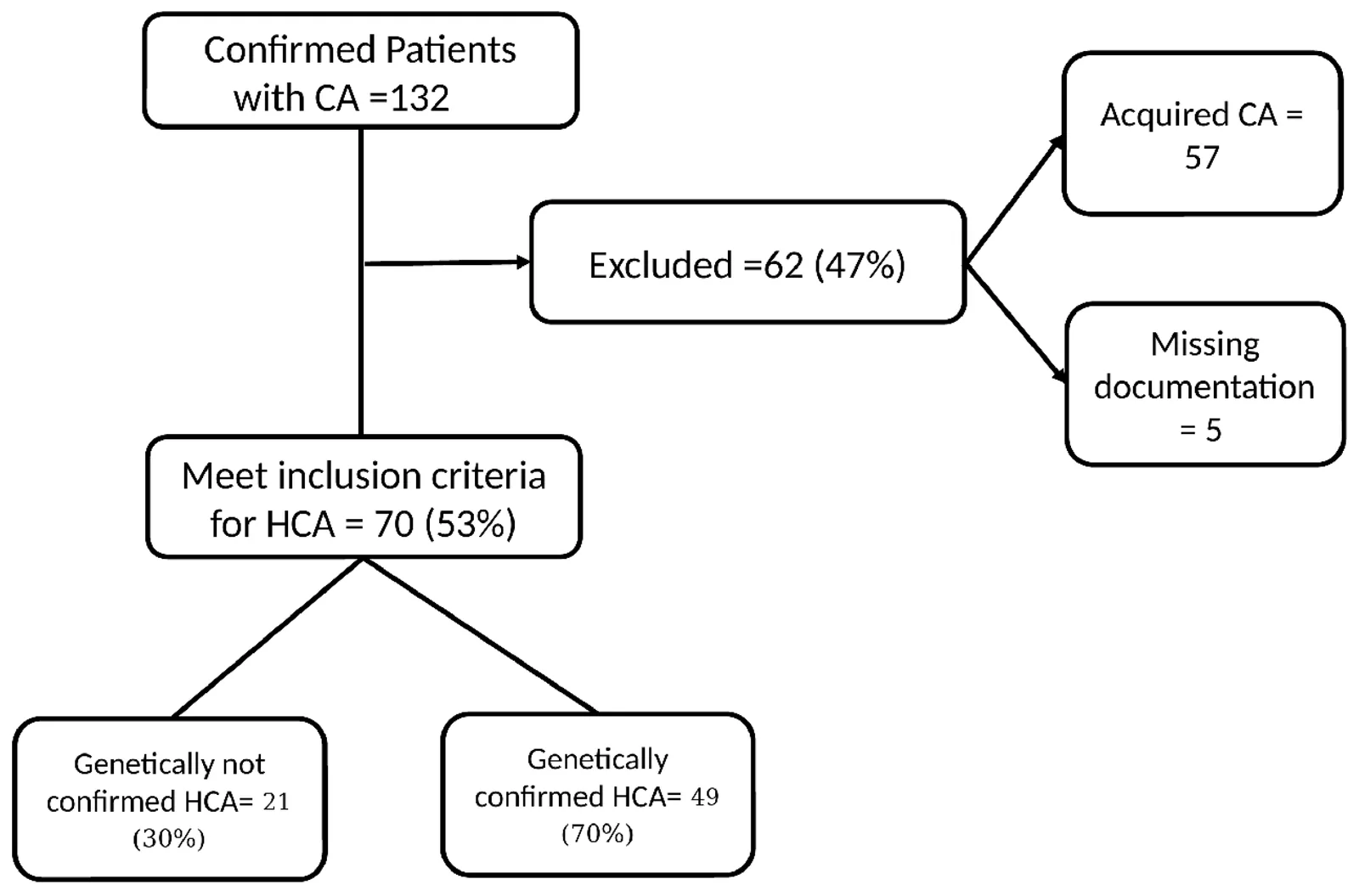
Flow Diagram of the Patients with Hereditary Cerebellar Ataxia. CA: Cerebellar ataxia, HCA: Hereditary cerebellar ataxia. Diagnostic criteria of Hereditary Ataxia (HA): Abnormal neurological examination with detection of clinical signs that are usually present in HA, AND exclusion of non-genetic causes of ataxia. Confirming the hereditary nature of the ataxia by 1—A pathogenic causative variant in agreement with the inheritance pattern was identified; 2—Identifiable phenotype that is characteristic of hereditary ataxia; 3—Family history of 2 or more similarly affected with a recognizable inheritance pattern.

A total of 132 patients were identified to have cerebellar ataxia from January 2012 until January 2022. Fifty-three percent (70/132) of our cohort met the inclusion criteria for HCA, while 47% (62/132) were excluded; among those, five patients were excluded due to insufficient documentation to distinguish between acquired and hereditary ataxia. Additionally, 57 patients were excluded because an acquired cause of ataxia was identified (Figure 1). Fifty-four percent (38/70) were females, and 45.7% (32/70) were males. The mean age at presentation was 56 months (range: 1.5–192 months, median: 42 months), while the mean age at diagnosis was 87.7 months (range: 12–192 months, median: 84 months).

Forty-nine of the 70 patients (70%) included in the study had genetically confirmed ataxia, while 30% (21/70) had no molecular genetic confirmation (Figure 1). Of the 70 patients, 12 (17.1%) were diagnosed with ATX-ATM, 5 (7.1%) with autosomal recessive spastic ataxia of Charlevoix-Saguenay (ATX-SACS), 4 (5.7%) with ATX-FXN, 3 (4.3%) with spinocerebellar ataxia type 2, and 3 (4.3%) with Joubert syndrome. Two patients were diagnosed with primary coenzyme Q10 deficiency type 4 (COQ10D4) (2.9%). Both patients received CoQ10 supplementation and had reported clinical improvement during follow-up, including reduced ataxia, clumsiness, and intentional tremor. Table 1 shows the remainder of the other diagnoses (Table 1).

**Figure 2:**
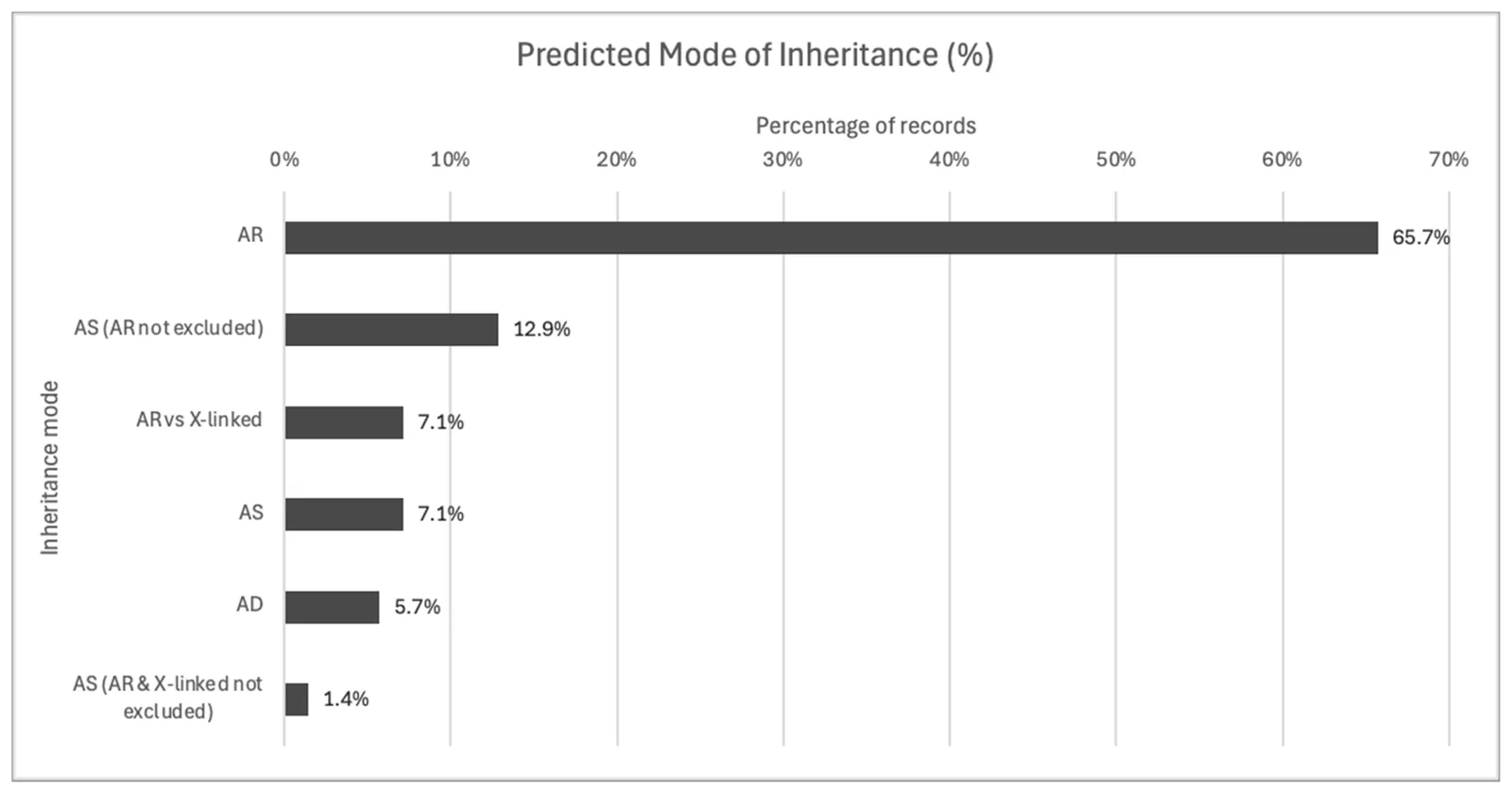
Classification of Patients According to Mode of Inheritance. Abbreviations: AR; Autosomal Recessive, AD; Autosomal Dominant, AS; Apparently Sporadic.

With respect to the mode of inheritance, ARCA was the most common (65.7%), followed by AS (AR not excluded) at 13% and AS and AS/X-linked at 7% each. Figure 2 shows a representation of the mode of inheritance.

There was no difference in the consanguinity rate between those patients in the genetically confirmed and not genetically confirmed groups (*p* = 0.719). Positive family history was significantly higher in the genetically confirmed group (*p* = 0.003).

### 3.2. Clinical Features

Fifty-five percent (38/69) of our patients had developmental delay, 22.9% (16/70) had seizures, and 7.1% (5/70) had epileptic encephalopathy. Fifty-three percent (32/70) had eye examinations. Among these 32 patients, 9.3% (3/32) had optic atrophy, 12.5% (3/32) had pigmentary retinopathy, 21.9% (7/32) had conjunctival telangiectasia, 21.9% (7/32) had nystagmus, 12.5% (3/32) had maculopathy, and 3.1% (1/32) had gaze palsy, ptosis, and rod-cone dystrophy. Twenty-one percent (13/61) reported having visual impairment. Hearing assessment was performed on four patients, and all of them were reported to have sensorineural hearing loss. Only 12.8% (9/70) of patients had an echocardiogram, and none of them had cardiomyopathy. A nerve conduction study (NCS) was performed for 31 patients—axonal polyneuropathy was found in 9 patients (9/31) (29%), and 70.1% (22/31) had normal NCS with no evidence of neuropathy. Other features reported for our cohort were choreoathetosis (2/70, 2.9%), tremors (5/70, 7.1%), and dystonia (1/70, 1.4%).

No statistically significant differences in clinical features were observed between the genetically confirmed and non-genetically confirmed groups (Table 2).

### 3.3. Genetic Testing Findings 

Sixty-two patients (88.6%) underwent molecular genetic testing during the study period. All genetic testing was outsourced to accredited clinical laboratories. Among these patients, 32/62 (51.6%) underwent whole-exome sequencing (WES), 13/62 (21.0%) underwent an NGS-based gene panel, 15/62 (24.2%) underwent targeted variant analysis due to a known familial variant or an affected family member with a similar phenotype, and 2/62 (3.2%) underwent chromosomal microarray analysis. Two patients underwent both WES and an NGS panel; for the purpose of calculating diagnostic yield by testing modality, these patients were included in the WES group and not in the panel group. Thus, each patient was assigned to a single testing category to avoid double counting.

The yield of the panels was 12/13 (92.0%), whereas the yield of WES was 21/32 (65.6%). Overall, a molecular diagnosis was established in 49/62 (79.0%) patients who underwent genetic testing.

### 3.4. Neuroimaging Findings

**Figure 3:**
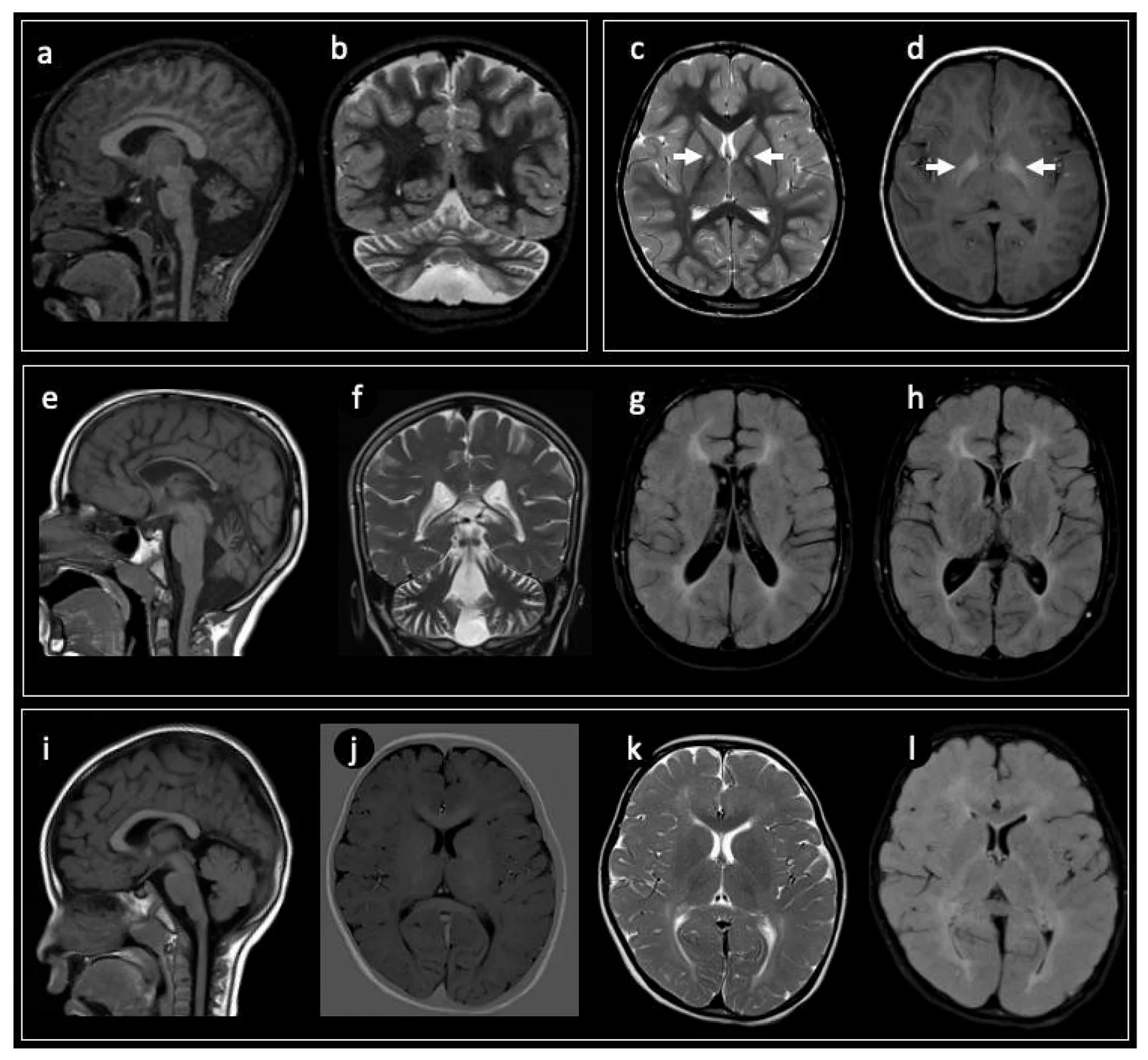
(**a**,**b**) *Ataxia telangiectasia:* A sagittal T1-weighted image (**a**) and coronal T2-weighted image (**b**) show isolated cerebellar atrophy. (**c**,**d**) *Pantothenate kinase-associated neurodegeneration (PKAN):* Axial T2-weighted (**c**) and axial T1-weighted (**d**) images show bilateral symmetric T2 hyperintensity and intrinsic T1 hyperintensity in the globus pallidus (arrows). (**e**–**h**) *Neuronal ceroid lipofuscinoses type 6:* Sagittal T1 (**e**) and coronal T2 (**f**) images demonstrate diffuse cerebellar atrophy and corpus callosum atrophy with mild cerebral atrophy, while axial FLAIR images (**g**,**h**) show periventricular and deep white matter hyperintensities. (**i**–**l**) Primary Coenzyme Q10 Deficiency Type 4: Sagittal T1 (**i**), axial T1 with inversion recovery (**j**), axial T2 (**k**), and axial FLAIR (**l**) images demonstrate symmetrical faint T2 and FLAIR hyperintensities and reduced T1 signal intensities in the cerebral white matter, without cerebral or cerebellar atrophy, in a 3-year-old patient.

**Figure 4:**
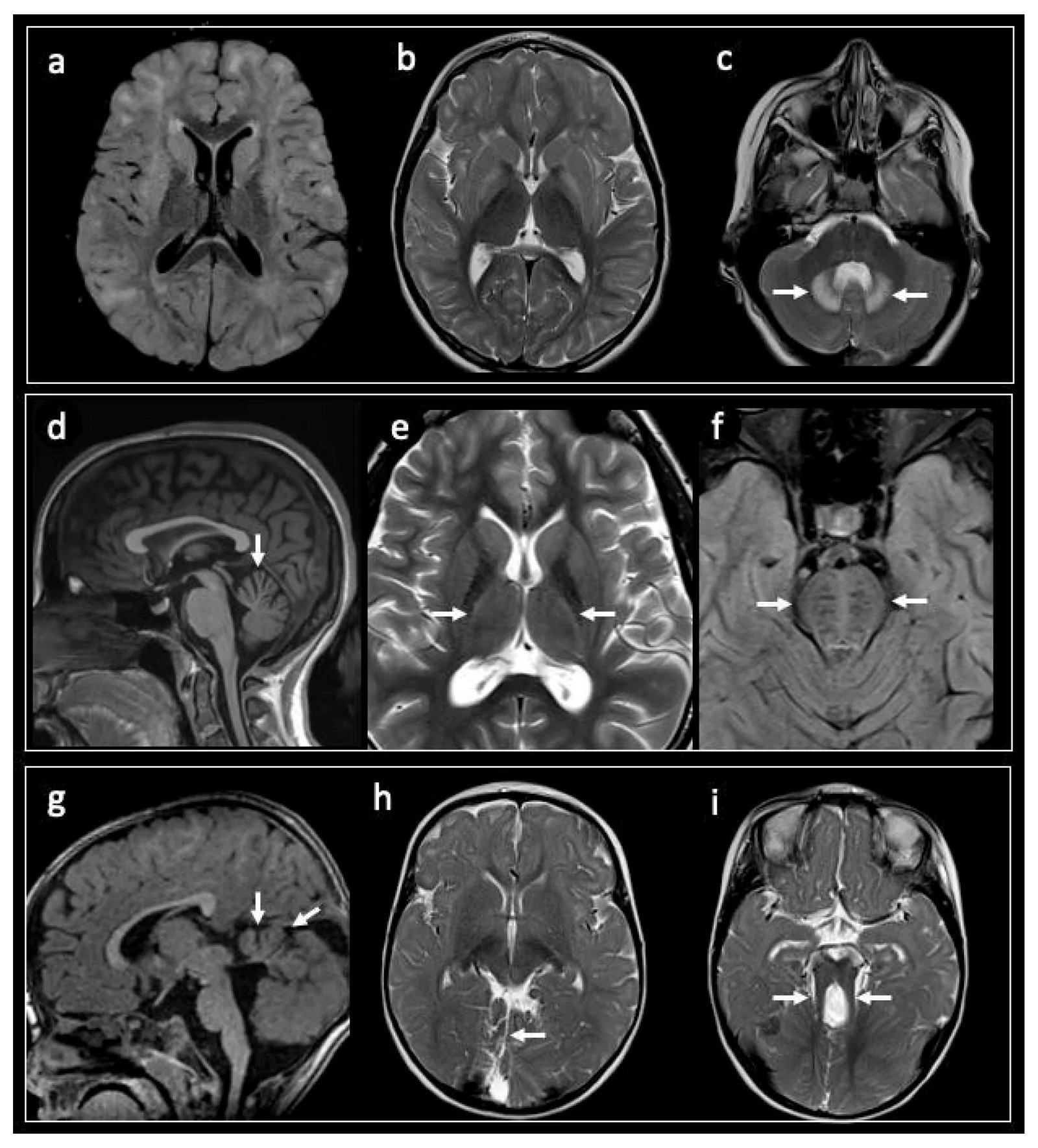
(**a**–**c**) L-2-hydroxyglutaric aciduria: Axial FLAIR images (**a**) show extensive white matter hyperintensities involving primarily subcortical white matter and U fibers. Axial T2-weighted images (**b**,**c**) also show involvement of the basal ganglia and dentate nuclei (arrows in (**c**)). (**d**–**f**) Autosomal recessive spastic ataxia of Charlevoix-Saguenay (ARSACS): Sagittal T1-weighted image (**d**) shows atrophy of the superior vermis (arrow) and inferior vermis part of the vermis is spared with relatively large pons. Axial T2 image (**e**) demonstrates T2 hyperintense rim around the thalami (arrows). Axial FLAIR image (**f**) shows the typical striated appearance of the pons (arrows). (**g**–**i**) Joubert syndrome: A sagittal T1-weighted image (**g**) shows small dysplastic vermis (arrows). Axial T2 images (**h**,**i**) demonstrate respectively median cleft of the vermis (arrow in (**h**)) and characteristic “molar tooth” sign with elongated superior cerebellar peduncles (arrows in (**i**)).

A total of 53 patients out of 70 (75.7%) underwent neuroimaging with brain magnetic resonance imaging (MRI). MRI scans of 42 patients were available for review, while the imaging of the remaining 11 patients was inaccessible. Out of the 42 patients whose MRIs were analyzed, 20 had normal scans. Cerebellar atrophy was the most frequent imaging finding (15/42, 35.7%). Cerebellar hypoplasia was present in two patients, one with chromosome Xq28 duplication syndrome and another with an indeterminate diagnosis. Brainstem atrophy was present in seven patients (16.7%). Cerebral white signal abnormalities were seen in nine patients, and two of them had a diffuse hypomyelination pattern. Atrophy of the spinal cord was present in one patient for whom no pathogenic variant was identified. The neuroimaging findings are summarized in Table 3, and Figure 3 and Figure 4 depict the representative images of selected patients.

## 4. Discussion

In the present study, we examined the largest cohort of patients with HCA in Oman. During the study period, 53% of patients presenting with cerebellar ataxia had HCA. Estimating the prevalence of ataxia from the literature was challenging due to the heterogeneity of these disorders, which encompasses diverse genetic etiologies, clinical presentations, and disease progressions. Studies on the prevalence of HCA have been conducted across various ethnic groups, revealing differing prevalences and variations in the most common types of HCA. A 2014 systematic review and meta-analysis assessed the global distribution and prevalence of HCA and hereditary spastic paraplegia in 22 population-based studies. It estimated the overall prevalence of ADCA at approximately 2.7 per 100,000 people and ARCA at 3.3 per 100,000, with higher rates in Portugal, France, and Canada [7,13]. The higher reported rates of ARCA in these countries may reflect an actual increase in prevalence, although these rates also could be influenced by methodological factors, including the number and scope of genes analyzed during genetic screening, as speculated by the authors [13]. Ruano et al. highlighted significant regional heterogeneity due to genetic and methodological differences and estimated that about 1 in 10,000 people worldwide are affected by HCA or HSP [7]. The prevalence rates of ADCAs vary considerably across regions, with certain subtypes showing notably high frequencies in specific populations—for example, SCA3 (*ATXN3*) in Gosei, Japan (19.2 per 100,000) and SCA2 (*ATXN2*) in Holguin, Cuba (47.9 per 100,000) [13].

Studies on hereditary ataxia from the Middle East and North Africa (MENA) remain limited, with most available data originating from Iran, Libya, Tunisia, and Saudi Arabia [14,15,16,17]. However, reports have varied from country to country, with a report of 7/100,000 in Saudi Arabia, 4.8/100,000 in Libya, and 4/100,000 in Tunisia (14–16). In the Gulf Cooperation Council (GCC), the current knowledge of HCA is largely based on case reports and case series, which reflects a lack of comprehensive epidemiological studies about these disabling disorders in highly consanguineous communities [18,19,20,21,22,23,24]. Using the total Omani population of 3,039,349 as a denominator, the crude estimate of the prevalence of ataxia in Oman is 4.34 per 100,000 and 2.3 per 100,000 for HCA using 70 as the enumerator for the total number of patients meeting the diagnostic criteria for HCA in this study [25]. With a more representative population study, the prevalence may not be significantly different than that reported in Saudi Arabia.

Previous studies have shown various mutation spectra across ethnicity and geographic distribution [13,17]. In the present study cohort, ATX-*ATM* was the most common HCA (17.1%), ATX-*SACS* was the second (7.1%), and ATX-*FXN* was the third (5.7%). ATX-*FXN* represents the most common form of hereditary ataxia globally, with an estimated prevalence of 2–4 per 100,000 individuals, a carrier frequency ranging from 1 in 60 to 1 in 100, and an incidence of approximately 1 in 29,000 [13,26,27]. However, in our cohort, ATX-*FXN* was the third most common subtype. This phenomenon could be explained by its relatively later age of onset compared to ATX-*ATM*, which typically presents in early childhood with a more aggressive course, immunodeficiency, and a higher risk of malignancy—features that often facilitate earlier diagnosis [28]. In contrast, ATX-*FXN* demonstrates considerable phenotypic variability, with 15–25% of cases showing milder symptoms, slower progression, and atypical features, often presenting after 25 years of age [29]. Together, these factors lead to underdiagnosis of *ATX-FXN* in the pediatric population. In Arab populations, the higher frequency of ATX-*ATM* may also be influenced by a founder effect [19]. Our cohort is not an exception, since ATX-*ATM* was also recognized as the second most prevalent form of ARCA in an Iranian cohort [17].

The landscape of hereditary cerebellar ataxias in the MENA region is highly diverse, although most available data originate from case reports and case series, with only a few large-scale studies conducted to date [14,15,16,17,18,19,20,21,22,23,24]. A recent Iranian study examining the genetic underpinnings of early-onset ARCA analyzed 162 patients and discovered pathogenic variants in 42 genes, which emphasizes the significant genetic heterogeneity of these disorders. *PLA2G6* or infantile neuroaxonal dystrophy (INAD) was the most frequent form of early ARCA, followed by ATX-*ATM*, ATX-*SACS,* and other SCA variants [17].

With respect to the GCC, there are reports of different types of hereditary ataxias, including but not limited to episodic ataxia, ataxia telangiectasia, ataxia with oculomotor apraxia, and other rare forms of HCA [18,19,20,21,22]. In a study investigating the phenotype and genotype of ATX-*ATM* in the Saudi population, 17 patients were analyzed, and 5 *ATM* variants were identified—3 previously known truncating variants and 2 novel compound heterozygous variants [19]. These findings highlight the genetic heterogeneity of hereditary ataxias and reinforce the importance of conducting large-scale studies to identify population-specific pathogenic variants and to optimize genetic testing strategies tailored to the regional genetic epidemiology. Furthermore, a recent report from Saudi Arabia has described a rare form of hereditary spinocerebellar ataxia in six affected individuals from a large consanguineous family, who were diagnosed with autosomal recessive cerebellar ataxia with spasticity caused by a homozygous variant in the *GBA2* gene [20]. Additionally, a founder effect was found within the Arab population of Saudi Arabia in the *RUBCN* gene, causing Salih ataxia [23,24].

Regarding local data on hereditary ataxias, even case reports on these heterogeneous disorders are scarce, and currently no available information exists for the genetic landscape of HCA at the national level [30,31]. Pratap-Chand et al. have reported 11 Omani children presenting with early progressive ataxia associated with sensorineural hearing loss. Although these patients were diagnosed with an autosomal recessive form of olivopontocerebellar atrophy with onset in infancy, the underlying genetic etiology was not identified in the study [30]. Another report described four affected Omani patients from two unrelated families who presented with progressive ataxia, cerebellar atrophy, disabling axonal sensorimotor neuropathy, hypercholesterolemia, and elevated serum alpha-fetoprotein levels. Genetic analysis identified biallelic variants in the *TDP1* gene as the underlying cause of ARCA in these patients [31]. To our knowledge, this study represents the first comprehensive clinical and genetic characterization of HCA in our population.

Despite major advancements in NGS, about 50% of clinically suspected ARCA cases remain genetically undiagnosed, which may be due to limitations of genetic testing, including deep intronic variants, complex rearrangements, novel genes or previously undiscovered etiological links, underreporting of variants of unclassified significance that may be relevant, and epigenetic changes [2]. Our cohort’s genetically confirmed ataxia percentage closely matched the findings of various studies [13,14]. The DY for molecular testing, including WES and panels, was 79%. However, we want to point out that clinical judgment has influenced the type of genetic testing chosen, and not all patients meeting the diagnosis of HCA underwent genetic testing; when testing was performed, the type of genetic testing chosen was variable.

The DY in the Cheng et al. cohort was 46.3% [32]. In this cohort of patients with autosomal recessive or sporadic cerebellar ataxia, WES was combined with copy number variant (CNV) analysis, illustrating how the use of complementary genetic testing approaches may influence diagnostic yield [32]. On the other hand, Mahdieh et al. reported that out of 162 patients from various ethnic groups in Iran, pathogenic variants were identified in 97 families, spanning 42 genes, which results in a DY of 59.9% [17]. In a comprehensive systematic review of next-generation sequencing (NGS) tests—targeted panels (TP), WES, and genome sequencing (GS)—for hereditary ataxia (HA), which included 33 studies with 3144 participants from diverse geographic locations and various HCA phenotypes, the median DY was 43–47.5%, with a wide range (9.5% to 100%) depending on cohort selection and methods [33]. Higher DY was observed in cohorts with autosomal recessive inheritance, specific hereditary ataxia phenotypes, clinical features including spasticity and neuropathy, early or late disease onset, and a positive family history or parental consanguinity [33,34]. Furthermore, other studies have reported that patients with a cerebellar-plus phenotype may be more likely to receive a molecular diagnosis than those with isolated cerebellar ataxia [35]. In addition, DY was slightly higher with targeted panel sequencing (46%) than with exome sequencing (41.9%), largely due to coverage of disorders caused by trinucleotide repeat expansions, although exome sequencing offers the advantage of detecting rare genes not covered by targeted panels [33]. These findings highlight that differences in cohort composition and the genetic testing strategy should be considered when comparing diagnostic yields across studies.

The relatively high DY achieved through both targeted gene panels and WES in this study may have been the product of a highly selected and unavoidably biased patient cohort for testing, reflecting the nature of the inclusion criteria, improved clinical characterization, and the timing of the study relative to the previously characterized genes, and the relative diagnostic impact of a positive family history compared to other study populations. In addition, our cohort was enriched for early-onset and familial disease and had a high rate of consanguinity (83%), which may have increased the likelihood of identifying biallelic pathogenic variants. Furthermore, our cohort included genetic disorders in which ataxia forms part of a broader neurodevelopmental, metabolic, neurodegenerative, or multisystem phenotype, some of which may be more readily identifiable through phenotype-guided genetic testing. These cohort characteristics may, in part, explain the higher diagnostic yield observed in our study. Conversely, the inclusion of sporadic and previously unexplained cases in other cohorts may contribute to lower diagnostic yields, despite the use of more comprehensive genomic analyses [32].

The relatively high diagnostic yield in our cohort also has important clinical implications, as molecular diagnosis may identify potentially treatable conditions. Two patients (2.9%) were diagnosed with COQ10D4 and received CoQ10 supplementation, with reported clinical improvement during follow-up, including reduced ataxia, clumsiness, and intention tremor. Although causality cannot be established from this retrospective observation, these cases illustrate the potential value of molecular diagnosis in identifying treatable neurometabolic disorders. Therefore, early recognition of potentially treatable neurometabolic and movement disorders remains important because timely molecular diagnosis may have implications for management and prognosis.

In our study cohort, the AR mode of inheritance was the most prevalent, accounting for 66%. This result may reflect the high rate of consanguineous marriages among our participants [8,9,36]. Moreover, ARCA is generally the most common HCA category in the pediatric age group [27]. Additionally, autosomal dominant (AD) spinocerebellar ataxias are typically late-onset, which explains the lower prevalence of ADCA in our cohort [37].

With regard to neuroimaging, cerebellar atrophy was the most common finding in our cohort (15/42, 35.7%), which is consistent with previous reports in the literature [38]. Although cerebellar atrophy is non-specific and can present in variable types of HCA [11,38,39], the presence of other neuroimaging findings in addition to cerebellar atrophy would be helpful for guiding a diagnosis.

To our knowledge, the present study is the first to document the frequency of HCA in Oman and the first to describe the different genotypes and phenotypes in our population. However, the study is single-centered, and the methodology is retrospective in nature. Thus, the collected data and the performed analysis might misrepresent the Omani population and render some of the results as statistically insignificant or not generalizable to this population. Another limitation of this study is the lack of systematic biochemical evaluation for acquired and potentially treatable causes of ataxia, including gluten ataxia and vitamin deficiencies, as well as other relevant laboratory investigations such as AFP, albumin, and cholesterol levels, limiting our ability to assess their diagnostic contribution. Nevertheless, this study could pave the way to establishing a diagnostic algorithm for HCA at the national or regional level due to the information it adds to the HCA landscape in this unique region.

## 5. Conclusions

This study provides the first comprehensive description of HCA in Oman and establishes an important foundation for understanding its genetic and phenotypic spectrum. ATX-*ATM* emerged as the most common subtype in our cohort. The insights from this study into the regional genetic landscape may help develop population-specific diagnostic guidelines and select molecular testing strategies for ataxia, especially in Oman and other populations with high consanguinity and distinct founder effects. Early recognition and molecular diagnosis of neurometabolic and movement disorders presenting with ataxia are important for timely management and prognosis, as illustrated by the potentially treatable neurometabolic disorders identified in our cohort. By characterizing the clinical, radiological, and molecular features of HCA, our findings contribute valuable data to regional and global efforts and may support the establishment of national registries, population-based screening programs, and future research into novel genetic causes.

## Figures and Tables

**Table 1:** Patients with a Confirmed Molecular Diagnosis of Hereditary Cerebellar Ataxia.

Diagnosis	*n* (%)
Ataxia Telangiectasia (ATX-ATM)	12 (17.1)
Charlevoix-Saguenay (ATX-SACS)	5 (7.1)
Friedreich Ataxia (ATX-FXN)	4 (5.7)
Joubert Syndrome	3 (4.3)
Familial Spinocerebellar Ataxia Type 2	3 (4.3)
Neuronal Ceroid Lipofuscinoses Type 6	2 (2.9)
Primary Coenzyme Q10 Deficiency Type 4 (COQ10D4)	2 (2.9)
Intellectual developmental disorder, autosomal recessive 57	2 (2.9)
AR L-2-hydroxyglutaric aciduria	2 (2.9)
Autosomal Recessive Spinocerebellar Ataxia-14 (SCAR14)	1 (1.4)
Autosomal Dominant Episodic Ataxia Type 6	1 (1.4)
Autosomal Recessive Bartter Syndrome Type 3	1 (1.4)
Brain–Lung–Thyroid Syndrome	1 (1.4)
Chromosome Xq28 Duplication Syndrome	1 (1.4)
DRPLA	1 (1.4)
Early Onset Progressive Encephalopathy with Brain Edema and Leukoencephalopathy Type 1	1 (1.4)
Familial Paroxysmal Kinesigenic Dyskinesia	1 (1.4)
Nicolaides-Baraitser Syndrome	1 (1.4)
Hereditary Spastic Paraplegia 12	1 (1.4)
Pantothenate Kinase-Associated Neurodegeneration (PKAN)	1 (1.4)
Spastic ataxia 8, autosomal recessive, with hypomyelinating leukodystrophy	1 (1.4)
Spinocerebellar Ataxia with Axonal Neuropathy Type 2	1 (1.4)
Unverricht–Lundborg Disease	1 (1.4)
Total	49 (70.0)

Abbreviations: Values are presented as n (%); DRPLA = Dentatorubral–Pallidoluysian Atrophy; AR = Autosomal Recessive. Percentages may not sum exactly to 70% due to rounding.

**Table 2:** Comparison of Clinical Features Between Genetically Confirmed and Non-Genetically Confirmed Groups.

Variable	Available Data (n/N) %	Genetically Confirmed (n = 49)	Non-Genetically Confirmed (n = 21)	*p*-Value
Developmental Delay	38/69 (55)	27/48	11/21	0.790
Seizures	16/70 (22.9)	12/49	4/21	0.615
Epileptic Encephalopathy	5/70 (7.1)	4/49	1/21	0.585
Visual Impairment	13/61 (21.3)	9/46	4/15	0.911
Eye Exam Abnormalities	32/60 (53.3)	25/44	7/16	0.141
Sensorineural Hearing Loss	4/66 (6)	2/46	2/20	0.395
Cardiomyopathy	0/9 (0)	0/9	0	**-**
GI Findings	3/69 (4.3)	3/48	0/21	0.135
Peripheral Neuropathy (NCS)	9/31(29)	7/20	2/11	0.429
Other Clinical Features				
Choreoathetosis		2	-	
Dystonia		1	-	
Tremors		3	2	

Values are presented as n (%) unless otherwise specified. NCS = Nerve Conduction Study; GI = Gastrointestinal. n/N represents the number of patients with the specified clinical feature (n) among those with available data for that variable (N). Percentages were calculated using N as the denominator; therefore, denominators vary between clinical variables because of missing data.

**Table 3:** Summary of MRI Findings in Patients of HCA.

Neuroimaging Findings	Patients with MRI (n = 42)
Cerebellar atrophy	15
Cerebellar hypoplasia	2
Cerebellar T2 hyperintensity	4
- White matter	2
- Nuclei	2
Brainstem	
- Atrophy	7
- Malformation	1
- Signal abnormality	5
Cerebral atrophy	7
Cerebral white matter signal abnormality	9
- Deep or periventricular white matter	5
- Subcortical white matter	2
- Corticospinal tracts	2
- Diffuse	2
Basal ganglia	
- Atrophy	1
- T2 hyperintensity	3
Spinal cord atrophy	1

Abbreviations: HCA: Hereditary cerebellar ataxia.

## Data Availability

The data supporting the findings of this study are available from the corresponding author upon reasonable request, subject to ethical and privacy restrictions.
